# Paternal grandmother’s smoking in pregnancy is associated with extreme aversion to bitter taste in their grandchildren

**DOI:** 10.1093/eep/dvac003

**Published:** 2022-02-16

**Authors:** Jean Golding, Marcus E Pembrey, Steven Gregory, Matthew Suderman, Yasmin Iles-Caven, Kate Northstone

**Affiliations:** Population Health Science, Bristol Medical School, University of Bristol, Bristol, UK; Population Health Science, Bristol Medical School, University of Bristol, Bristol, UK; Population Health Science, Bristol Medical School, University of Bristol, Bristol, UK; Population Health Science, Bristol Medical School, University of Bristol, Bristol, UK; Population Health Science, Bristol Medical School, University of Bristol, Bristol, UK; Population Health Science, Bristol Medical School, University of Bristol, Bristol, UK

**Keywords:** ALSPAC, supertasters, bitter taste, PROP, transgenerational, grandmother smoking in pregnancy, paternal grandmother

## Abstract

Although there are many examples in the experimental literature of an environmental exposure in one generation impacting the phenotypes of subsequent generations, there are few studies that can assess whether such associations occur in humans. The Avon Longitudinal Study of Parents and Children (ALSPAC) has, however, been able to determine whether there are associations between grandparental exposures and their grandchildren’s development. Several of our studies, including sensitivity to loud noise, have shown associations between a grandmother smoking in pregnancy and the phenotype of the grandchild. These results were mostly specific to the sex of the grandchild and to whether the prenatal (i.e. during pregnancy) smoking occurred in the maternal or paternal grandmother. Here, we have used ancestral data on prenatal smoking among the grandmothers of the ALSPAC index children to examine possible effects on the grandchild’s ability to detect the bitter taste of PROP (6 *n*-propylthiouracil), distinguishing between the 10% deemed ‘extreme tasters’, and the rest of the population (total *N* = 4656 children). We showed that grandchildren whose paternal (but not maternal) grandmothers had smoked in pregnancy were more likely than those of non-smoking grandmothers to be extreme tasters [odds ratio (OR) 1.28; 95% confidence interval (CI) 1.03, 1.59] and that this was more likely in granddaughters (OR 1.42; 95% CI 1.03, 1.95) than grandsons (OR 1.18; 95% CI 0.88, 1.60). This pattern of association between paternal foetal exposure and the granddaughter’s development has been found with several other outcomes, suggesting that investigations should be undertaken to investigate possible mechanisms.

## Introduction

Although there is mounting evidence within animal species that environmental exposure to one generation can result in developmental changes in subsequent generations, little attention has been paid to the possibility of such intergenerational/transgenerational associations in humans. The academic groups that have been developing programmes to investigate such possibilities include ourselves. Such effects have variously been called ‘intergenerational, multi-generational, or transgenerational’ (see [1] for discussion of definitions). For simplicity, the term ‘transgenerational’ will be used throughout this paper.

Using data collected from the Avon Longitudinal Study of Parents and Children (ALSPAC), we have shown associations between the ‘paternal’ grandmother smoking in the pregnancy that resulted in the birth of the study father and outcomes that are more likely in the granddaughters than grandsons: namely asthma [2]; myopia before the age of 7 [3]; and greater fat mass in childhood, adolescence, and early adulthood [4–6]. Thus, there is evidence of sex-specific transgenerational associations in grandchildren when the paternal grandmother has smoked in pregnancy. In parallel-specific DNA, methylation changes have been shown in granddaughters (but not grandsons), whose paternal grandmothers smoked in pregnancy [7]; in early-onset myopia, these changes were particularly linked to genes known to be associated with myopia, thus indicating possible biological plausibility for the myopia association [3].

In parallel with the above, many of the associations demonstrated for ‘maternal’ grandmother smoking prenatally have also been sex-specific, including increased risk of high levels of two autistic traits in granddaughters [8], and increased foetal growth in grandsons [5]. However, there were also independent associations of ‘both’ maternal and paternal grandmothers smoking prenatally and increased weight in both grandsons and granddaughters during childhood, which were associated with lean rather than fat mass [5].

As part of our programme to investigate transgenerational associations with grandparental smoking, we selected two sensitivity traits that were measured in the ALSPAC participants (i.e. the grandchildren). Sensitivity to loud sounds at 6 years was associated with the maternal grandmother smoking in pregnancy and was increased in grandsons and reduced in granddaughters. More objectively, at age 11, the grandsons selected a lower volume when listening to music than the granddaughters, confirming that granddaughters were more tolerant of very loud sounds than the grandsons [9]. In the present study, we test the possibility that extreme sensitivity to a bitter taste is associated with environmental exposures in the grandparents’ generation. In particular, we test whether the smoking habits of any of the four grandparents maternal grandmother (MGM), maternal grandfather (MGF), paternal grandmother (PGM), and paternal grandfather (PGF) were associated with a grandchild’s extreme sensitivity to the bitter taste of 6 *n*-propylthiouracil (PROP)—and if so, whether the association is specific to the sex of the grandchild.

Bartoshuk [10] has described how the perception of the bitter taste of PROP can be measured along a continuous scale, with non-tasters at the low end, then increasing levels of taste sensitivity. Those at the extreme upper end are those who find the taste extremely unpleasant, almost intolerable. Twin studies have indicated a genetic component with a broad range of heritability estimates from 0.36 to 0.73 [11, 12], although family linkage studies have shown the heritability to be less than straightforward. There has been little study of epidemiological associations, although there is some evidence that the threshold of bitter taste differs in smokers compared to non-smokers [e.g. 13].

In this paper, we investigate whether the association between the grandchildren’s sensitivity to loud sounds and their paternal grandmothers’ prenatal smoking is mirrored by a similar association in grandchildren who are extreme tasters of PROP. We anticipate that if there is an association, as with the sensitivity to loud sounds, the effect sizes will differ between the sexes of the grandchildren.

## Material and Methods

### The Study Sample

ALSPAC is a pre-birth cohort designed to determine the environmental and genetic factors that are associated with health and development of the study offspring [14–16]. Pregnant women who were residents of Avon, UK, with expected dates of delivery between 1 April 1991 and 31 December 1992 were recruited. The initial number of pregnancies enrolled was 14 541 (an estimated 80% of the eligible population). From these initial pregnancies, there was a total of 14 676 foetuses, resulting in 14 062 live births and 13 988 children who were alive at 1 year of age. Data were collected at various time points using self-completion questionnaires, biological samples, hands-on measurements, and linkage to other data sets. Full details of all the data collected are available on the study website: www.bristol.ac.uk/alspac/researchers/data-access/data-dictionary/. Ethical approval for the study was obtained from the ALSPAC Ethics and Law Committee (ALEC; IRB00003312) and the Local Research Ethics Committees [17]. Detailed information on the ways in which confidentiality of the cohort is maintained may be found on the study website: http://www.bristol.ac.uk/alspac/researchers/research-ethics/

ALEC agreed that consent was implied if questionnaires were returned. Informed written consent was obtained for all biological samples prior to analysis and for certain invasive procedures during the hands-on assessments (which were optional to attend) from the participant and/or legal guardian. All study methods were performed in accordance with relevant guidelines and regulations. Together with the local Health Services ethics committees, ALEC has approved the linkage of the DNA and methylation data to the detailed assessments and other information on the parents and children. Analyses of biological samples, including genetic and DNA methylation, are only carried out for individuals for whom informed generic consent has been received.

As part of the study design, there was a concerted effort before the child’s birth to obtain from each of the parents (G0) details of their own parents (G0^p^). The pregnant women were sent four questionnaires during pregnancy, one of which requested details of their parents; in parallel, they were sent two questionnaires for their partners to complete, one of which included similar questions concerning the partner’s own parents.

### Information on the Study Child’s Grandparents

The questionnaires sent to the parents (G0) during pregnancy elicited information on the following items of relevance to this project:

- The smoking histories of each of their own parents (i.e. the study grandparents (G0^p^)).
- If parents had reported that their mothers (G0^p^) had smoked, they were each asked whether their mothers had smoked when pregnant with them—and, if so, were given the responses ‘yes/no/don’t know’, from which to select. Thus, the parents who replied ‘don’t know,’ had a mother who smoked but the parent was unsure whether she had smoked during her pregnancy. As with our other studies, we have analysed these data assuming that these women did smoke during pregnancy [4, 5, 18]. This assumption was strengthened by demonstrating that the mean birthweights of this group of study mothers were reduced by ∼200 g when compared with those mothers who reported that their mother had definitely not smoked in pregnancy [4].
- The ages of each grandparent (G0^p^) when the study parent (G0) was born.
- For each of the four study grandparents (G0^p^), their years of birth were estimated from details of their ages at the time of birth of the relevant parent and the age of the parent when the study child was born.
- Two variables are based on the annual real gross domestic product (GDP) per capita at the time of the birth of each grandparent. This is based on the total amount produced in the UK in a year, per inhabitant, and corrected for inflation. ‘Corrected for inflation’, here means that everything is expressed in terms of 1990 US dollars. The annual real GDP per capita comes from Maddison ( [19]; updated 2013) who provided internationally comparable historical macro-economic time series of such variables. Somewhat loosely, the annual real GDP per capita indicates the average economic activity per inhabitant each year. We have used both the annual real GDP decomposed into a trend and a business cycle. The trend captures long-run trends in economic activity. The cycle is the business cycle fluctuating along the trend. The decomposition is the Hodrick–Prescott filter with smoothing parameter 100 over the years 1835–2001. The sum of trend and cycle equals the original variable.
- The social class of each grandparent (based on a categorization of their occupations as reported by their offspring, the study parents (G0)).
- The educational qualifications of each grandparent (grouped into two categories, according to their completion of national examinations or their equivalent).
- Ethnic group (grouped as white and all other).
- Parity (for grandmothers only)—i.e. whether the study parent was the first or later birth to that grandmother.

These variables were considered as possible transgenerational candidates and/or possible confounders.

### Testing the Child’s Perception of the Taste of PROP

Paper disks impregnated with PROP have been shown to be a crude but rapid way to test responses to PROP in large groups [20]. As part of a face-to-face clinic session held when the children were aged 10 years, a nutritionist interviewed them about their diet. Following this, the nutritionist proceeded to assess the subject’s reaction to a bitter (PROP) challenge using a general visual analogue scale (gVAS) [21].

The disks were prepared by soaking circular pieces of filter paper (Whatman #1) in a saturated solution of PROP (at near-boiling temperature) and then drying them. The PROP crystallizes into the paper, thus allowing the paper to serve as a convenient way to permit a subject to taste a limited quantity of PROP crystals. The PROP crystals go into solution in the subject’s saliva and produce a high concentration of PROP at the taste receptor sites. The paper produces bitterness approximately equivalent to a solution of 0.0032 M, close to the highest concentration of PROP that will remain in solution when PROP solutions are refrigerated for storage. The purpose of using a high concentration for screening is that PROP functions for non-tasters, medium tasters, and supertasters diverge; thus, the highest practical concentration of PROP produces the most accurate assessment.

In a clinic setting, a research interviewer explained to the 10-year-old child that they were going to taste a piece of paper and would then be asked to mark how strong the taste was on a line. To explain the concept, the child was asked to describe the loudest, most intense sound they had ever heard. The interviewer then pointed to a 10-cm line on the datasheet, explaining that the left-hand end of the scale represented no sensation and the right hand end the most intense sensation. Once the child had grasped the concept, the child was asked to place the impregnated paper on the tongue and move it around for about 10 seconds. They were then asked to make a mark on the line to indicate how intense a sensation it was: 0 measured no taste at all, and 10 the strongest taste. Test–retest reliability was assessed on a randomly chosen 168 children who were tested an average of 33 days later. The correlation between the two test scores was 0.62 (*P* < 0.0001) [22].

The testing was not carried out when the child was fasting. No record was kept as to when the child had last eaten or what had been eaten. The test was carried out part way through a set of different exercises and interviews, and the child would not have been able to eat immediately before being tested. Half the tests occurred in the morning (from 9.30 am) and half in the afternoon.

**Figure 1:**
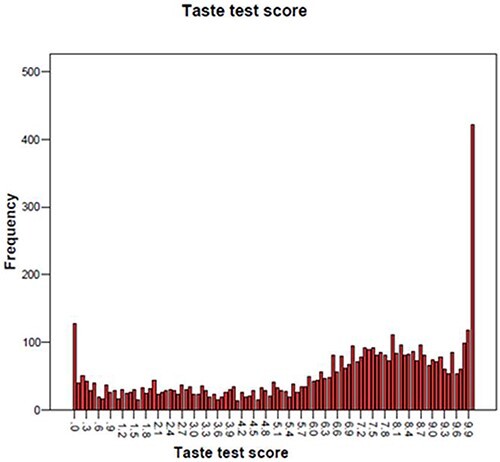
The distribution of the taste test scores at age 10

Using the data collected (Fig. 1), we identified as predicted a group of children who scored low on the test, then a roughly normal-shaped curve that indicated those who could taste to various degrees, and then an extreme group who did not conform to the normal curve. Those children who scored ≥9.9 we have named ‘extreme tasters.’

### Statistical Approach

We analysed the data in a hypothesis-free structure, taking care to ensure that we avoided Type I errors as much as possible (and, therefore, we did not allow for multiple testing). Based on our own studies and previous results from the literature, we hypothesized that if there were effects of a transgenerational nature, the associations would differ between the sexes.

First, each maternal and paternal inheritance line was analysed separately. Potential confounders were identified as variables with unadjusted associations of *P* < 0.05; backward stepwise logistic regression analyses were employed with the outcome as extreme tasters. Second, the variables surviving the analyses for each maternal grandparent were combined and offered again to backwards stepwise analysis; this was repeated for the paternal grandparents. Third, a final analysis combined all data remaining in the second step. The data are presented as unadjusted and adjusted odds ratios (ORs) with their 95% confidence intervals (CIs). Differences between the sexes compared the adjusted ORs and CIs for each sex.

## Results

### The Taste Test Score

The 10-year taste test was carried out in 5294 of the 7442 (71%) children that attended the clinic at this age. The test was not carried out for the remainder primarily due to the appropriate impregnated paper disks being unavailable. The children who did not do the test did not differ from those who did by sex, maternal age, or social characteristics.

As shown in other studies, there is an unusual distribution of scores (Fig. 1), with a steady frequency for the first half of the scores (the non-tasters), then a normal distribution between scores of 5 and 9.8 (the tasters), and then a sharp increase in frequency for the scores of 9.9+ (the extreme tasters). Thus defined, the prevalence of extreme tasters was 10.2% in this cohort. It should be noted that this group differs from those generally named ‘supertasters,’ which have been generally defined as the top quartile of the distribution, rather than the top decile as defined here.

## The Maternal Grandparents

### Unadjusted Associations

The ways in which characteristics of the maternal grandparents (MGM and MGF) were associated with their grandchild’s extreme taster status are shown in the left-hand columns of Table 1. The data demonstrate statistically significant trends with the MGM and MGF years of birth (the more recently the grandparent had been born, the more likely the grandchild was to be an extreme taster), as well as similar trends with the national GDP at the year of birth of the grandparents (the higher the GDP, the greater the risk). There were also differences in the rate of tasters with the maternal grandparents’ education levels (grandparents with higher qualifications were less likely to have extreme taster grandchildren compared to those with lower qualifications). There was a similar finding with social class (maternal grandparents in the lower social classes, based on their occupations, were more likely to have grandchildren who were extreme tasters compared to those with higher social classes). There were no associations with smoking, age at birth of the parent, ethnic group, or the grandmother’s parity at the birth of the parent.

**Table 1:** Proportion (*n*) of grandchildren who were extreme tasters at age 10 for each feature of their grandparents

Variable	MGM	MGF	PGM	PGF
*Year of birth*				
Pre-1925	8.5% (44)	8.3% (68)	8.6% (46)	8.6% (69)
1925–1929	8.2% (54)	7.6% (63)	8.7% (45)	7.2% (39)
1930–1934	8.6% (85)	10.9% (102)	8.2% (48)	8.6% (47)
1935–1939	9.4% (98)	9.1% (79)	9.8% (50)	11.1% (45)
1940–1944	11.8% (91)	11.4% (58)	10.0% (36)	8.1% (20)
1945+	12.3% (57)	13.1% (30)	9.3% (11)	*x*
*P*	0.002**	0.006**	0.385	0.483
*N*	4429	4177	2627	2534
*P* for mean YOB	0.006**	0.003**	0.218	0.668
*P* for mean GDP	0.003**	0.004**	0.199	0.441
*P* for business cycle	0.575	0.614	0.875	0.291
*Ethnic background*				
White	10.1% (476)	10.1% (471)	9.5% (358)	8.5% (352)
Non-white	6.8% (5)	8.5% (7)	13.6% (11)	15.5% (15)
*P*	0.342	0.646	0.219	0.047*
*N*	4770	4755	3854	3844
*Education level*				
Higher	8.1% (111)	8.0% (109)	7.2% (70)	8.3% (95)
Lower	10.8% (245)	10.5% (219)	9.5% (188)	9.6% (177)
*P*	0.010**	0.018*	0.031*	0.215
*N*	3637	3447	2949	2983
*Ever smoked*				
Yes	10.2% (252)	10.5% (359)	9.7% (211)	8.9% (251)
No	10.2% (224)	9.0% (109)	9.1% (151)	10.0% (76)
*P*	0.949	0.154	0.486	0.370
*N*	4674	4629	3835	3567
*Age at birth of parent*				
<25 years	10.5% (171)	10.0% (82)	9.0% (98)	8.8% (48)
25–34	9.0% (206)	10.0% (246)	9.7% (174)	8.8% (163)
35+	9.9% (52)	8.0% (72)	7.6% (34)	9.4% (75)
*P*	0.327	0.136	0.612	0.646
*N*	4429	4177	3317	3192
*Parity*				
0	9.5% (143)	-	8.1% (44)	-
1+	10.5% (340)		9.5% (86)	
*P*	0.143		0.375	
*N*	4823		1445	
*Smoked prenatally*				
Yes	10.7% (167)	-	10.7% (167)	-
No	9.9% (307)		8.6% (193)	
*P*	0.370		0.028*	
*N*	4654		3815	
*Social class*				
I	*y*	8.4% (29)	*y*	7.4% (20)
II	8.5% (66)	8.1% (94)	9.3% (55)	10.5% (100)
IIINm	8.8% (89)	9.1% (48)	8.2% (54)	9.3% (45)
IIIM	12.6% (15)	11.6% (195)	9.2% (10)	8.5% (133)
IV	11.0% (64)	8.8% (20)	9.7% (43)	8.9% (17)
V	13.5% (35)	10.4% (11)	8.6% (18)	8.7% (9)
*P*	0.007**	0.015*	0.883	0.479
*N*	2754	4051	2031	3553

*x* denotes combined with 1945+; *y* denotes combined with Social Class I; GDP = gross domestic product; MGM = maternal grandmother; MGF = maternal grandfather; PGM = paternal grandmother; PGF = paternal grandfather; YOB = year of birth.

* *P* < 0.05.

** *P* < 0.01.

### Adjusted Analyses

The four variables with unadjusted associations at *P* < 0.05 with the MGM’s socioeconomic circumstances were offered to the stepwise logistic regression—only one remained as independently associated—the trend in GDP present at the time of the MGM’s birth (OR 3.30; 95% CI 1.54, 7.05; *P* = 0.002).

For the MGF, the same four variables were offered to the stepwise logistic regression. Again, only one variable remained—the trend in year of birth of the grandfather (OR 1.02; 95%CI 1.01, 1.03; *P* = 0.003) per year.

## The Paternal Grandparents

### Unadjusted Associations

Only three variables concerning the paternal grandparents (PGM and PGF) were associated at *P* < 0.05 with having extreme taster grandchildren (Table 1): increased rates when the PGF was non-white; increased rates if the PGM had a lower level of education; and an increased rate if the PGM smoked prenatally when expecting the study father.

### Adjusted Association

The result of the stepwise analysis resulted in just one variable remaining—the PGM smoking prenatally (OR 1.28; 95% CI 1.03, 1.59; *P* = 0.028).

### The Final Model

Offering to a further stepwise regression, the three grandparental measures that remained (the GDP at the year of birth of the MGM, the actual year of birth of the MGF, and the PGM smoking in pregnancy), the GDP measure dropped out, and just two variables remained: the year of birth of the MGF (OR 1.02; 95% CI 1.01, 1.04; *P* = 0.002) and the prenatal smoking of the PGM (OR 1.28; 95%CI 1.01, 1.63; *P* = 0.045). It is of note that the effect size of each of these variables did not change on adjustment for the other. For the focus of this paper, the PGM’s prenatal smoking, the number in the analysis had dropped from 3811 when unadjusted, to 3284 on adjustment. Therefore, because the effect size did not change on adjustment, we have concentrated on the unadjusted variables when considering the possibility of sex differences since this enables the largest numbers for analysis to be included.

### Differences between Grandsons and Granddaughters

Table 2 presents the ORs for the grandchildren being extreme tasters according to whether a grandmother smoked and according to whether their own mother smoked. There was little difference in the risk of the grandchild being an extreme taster if the MGM had smoked during pregnancy—and there was no difference between the sexes. For the PGM who smoked prenatally, however, the granddaughters were more at risk than the grandsons (although the interaction was not significant). The increased odds sizes for the granddaughters, in comparison with the grandsons, was apparent whether or not their own mother herself had smoked during pregnancy.

**Table 2:** Associations between the odds of the grandchild being an extreme taster if the grandmother smoked whilst expecting the grandchild’s parent

		MGM			PGM	
Population tested	*N*	OR [95% CI]	*P*	*N*	OR [95% CI]	*P*
All	4656	1.10 [0.90, 1.34]	0.366	3816	1.28 [1.03, 1.59]	0.028*
Boys	2295	1.10 [0.84, 1.43]	0.504	1879	1.18 [0.88, 1.60]	0.267
Girls	2361	1.11 [0.82, 1.49]	0.509	1937	1.42 [1.03, 1.95]	0.033*
*M−*						
All	4005	1.04 [0.83, 1.30]	0.744	3347	1.25 [0.98, 1.58]	0.069
Boys	1975	1.05 [0.78, 1.42]	0.727	1649	1.17 [0.84, 1.61]	0.353
Girls	2030	1.03 [0.73, 1.44]	0.880	1698	1.37 [0.97, 1.94]	0.076
*M+*						
All	639	1.23 [0.77, 1.97]	0.376	454	1.40 [0.77, 2.53]	0.271
Boys	317	1.15 [0.61, 2.17]	0.668	224	1.21 [0.54, 2.72]	0.641
Girls	322	1.34 [0.68, 2.67]	0.398	230	1.68 [0.69, 4.10]	0.255

MGM = maternal grandmother; PGM = paternal grandmother; *M−* = mother did not smoke in pregnancy; *M*+ = mother did smoke in pregnancy.

* *P* < 0.05.

## Discussion

This work follows on from our previous studies concerning the grandchildren with sensory impairments, where we have shown sex-specific associations with prenatal smoking of one but not both grandmothers [3, 9]. Here, we have shown that an excessive sensitivity to a bitter taste is associated with prenatal smoking of the ‘paternal’, but not the ‘maternal’, grandmother. This result is particularly associated with granddaughters rather than grandsons. We have indicated that this appears to be independent of the social circumstances of the grandparents, of the years in which they were born, and of the actual smoking habit of the mothers of the children.

The participant’s response to PROP was used to identify their sensitivity to a bitter taste. The method used involved paper disks impregnated with the substance. Although crude, this has been shown to be an efficient way to test populations such as the children taking part in ALSPAC [22–24]. Test–retest correlation was observed to be *R* = 0.62 (*P* < 0.001).

This study has concentrated on investigating whether any aspects of the grandparents available to the study were associated with the extreme taster phenotype in their grandchildren. After multivariable analysis only two remained in the analysis: (i) the year of birth of the MGF (such that the more recently he was born the greater the risk of the grandchild being an extreme taster) and (ii) whether or not the PGM had smoked during the pregnancy when she was carrying the grandchild’s father. As in most of the studies where we have found an association with the PGM smoking prenatally, we found a sex difference in the grandchildren, with granddaughters being at a higher risk of being an extreme taster compared with grandsons.

We did not find any independent social differences specific to the extreme tasters. Although there were unadjusted associations with maternal grandparental education levels and social class characterization, these were secondary to the years in which the grandparents were born. This raises the question of what might explain such an association. One possible explanation concerns the changing composition of the environment, which may have had epigenetic effects on the successors of the grandparents. There are many potential environmental candidates that have changed in prevalence over time, from air pollution due to the combustion engine to electric or magnetic non-ionizing radiation.

We had no information on medications used during the pregnancies of the grandmothers who smoked. However, we did look at the common forms of illness in the grandparents, including diabetes, schizophrenia, depression, cardiovascular disease, and bronchitis, but there were no signs that these factors were associated in any way with our results.

Among the ALSPAC 10-year-old children, boys were more likely to be extreme tasters than girls (11.6% of boys vs 8.8% of girls; *P* < 0.001). There was also an association with maternal smoking such that mothers of extreme tasters were more likely themselves to have been smokers in early pregnancy (12.9%) compared with mothers of non-extreme tasters (9.6%; *P* = 0.003). To determine whether maternal smoking played any part in our findings, we stratified the data by prenatal smoking and grandchild’s sex. We found that there was little difference to the overall pattern (Table 2)—there was still an association with the prenatal smoking of the PGM but not of the MGM, and the ORs for the girls were greater than those for the boys when the PGM had smoked. This is in contrast to our study on sensitivity to loud sounds, where the association with PGM smoking in pregnancy was found more in the grandsons than the granddaughters [9].

As we noted elsewhere [6], when we discussed mechanisms by which smoking of the PGM might have an influence on the grandchild, smoking is an established cause of DNA damage of various kinds, including effects on sperm [25]. The DNA damage response system results in the DNA in the nucleus being less tethered/restrained, and this increased mobility may facilitate access to DNA repair complexes [26]. This ‘mobility,’ in turn, may compromise the control of DNA repeat sequences and transposable elements by DNA methylation and repressive chromatin states in the germline and, in turn, in the emerging nervous system of the early embryo of the next generations. Other theories concern differences in the cigarettes smoked by the grandmothers. For example, we do not know what proportion were likely to be mentholated, or indeed to contain pesticides such as DDT, known to have an epigenetic effect [27].

However, there is a further possibility: the father will have inherited one X chromosome from his mother (the study child’s paternal grandmother) and will have only passed that on to his daughters (her granddaughters) but not his sons (the study grandsons). This raises the possibility that an epigenetic effect involving the X chromosome may be involved. Intriguingly, it has recently been shown that the smoking of the paternal grandmother is, as predicted, associated with differences in DNA methylation at CpG sites on the X chromosome of the granddaughter but not the grandson [7].

Little is known about the mechanism linking extreme tasters with grandmaternal smoking during pregnancy; beyond that, it does not appear to be genetic [22]. If the non-genetic transmission is via the germline as hypothesized above, we would at least expect to observe these effects in the epigenomes and transcriptomes of extreme tasters, likely as metastable epialleles in a wide range of tissues [28]. A preliminary test of this hypothesis could, therefore, ask if DNA methylation or gene expression in a peripheral tissue differs in extreme tasters. Although this is currently unknown, there is evidence of associations between taste preferences and DNA methylation in peripheral blood [29, 30].

### Strengths and Limitations

This study has a number of strengths: (i) it is large relative to most studies concerning tasters; (ii) the children involved were based on a geographic population and were not selected in any way; and (iii) information on grandparental background was obtained by postal questionnaire from the parents and was not available to the fieldworkers carrying out the taste test 10 years later.

The major limitation to the results concerns the fact that the numbers of grandchildren involved in the analysis were relatively small in comparison, for example, with the numbers involved in the adverse reaction to loud noise where the prevalence of extreme reaction was similar at about 10% (*N* ∼4000 for taste vs ∼6000 for noise). A second limitation is the fact that we have no information on the genetics of the grandmothers in the study, which is known to have some effect on smoking behaviour and taste. Third, the results are specific to PROP, and cannot be assumed to be also true of other bitter tastes such as phenylthiocarbamide or quinine.

## Conclusion

As predicted, we have shown an association between one specific grandmother smoking during pregnancy and the odds of the grandchild being an extreme taster, especially if the grandchild was a granddaughter. These results should be investigated further either by comparing results with another cohort study (although we do not know of any that yet have measures of PGM smoking and grandchild’s taste test result) or by demonstrating a biological mechanism for our findings.

## Data Availability

ALSPAC data access is through a system of managed open access. The steps below highlight how to apply for access to the data included in this paper and all other ALSPAC data. Please read the ALSPAC access policy (http://www.bristol.ac.uk/media-library/sites/alspac/documents/researchers/data-access/ALSPAC_Access_Policy.pdf), which describes the process of accessing the data and biological samples in detail, and outlines the costs associated with doing so. You may also find it useful to browse our fully searchable research proposals database (https://proposals.epi.bristol.ac.uk/), which lists all research projects that have been approved since April 2011. Please submit your research proposal (https://proposals.epi.bristol.ac.uk/) for consideration by the ALSPAC Executive Committee using the online process. You will receive a response within 10 working days to advise you whether your proposal has been approved. If you have any questions about accessing data, please email: alspac-data@bristol.ac.uk.
